# Supplementary material for: Increased sensitivity in electron–nuclear double resonance spectroscopy with chirped radiofrequency pulses
Source: Magn Reson (Gott). 2025 Jan 24;6(1):33–42. doi: 10.5194/mr-6-33-2025 (PMC12247087; doi:10.5194/mr-6-33-2025)
Supplement: The supplement related to this article is available online at: https://doi.org/10.5194/mr-6-33-2025-supplement. [file mr-6-33-2025-supplement.pdf]

Supplement of Magn. Reson., 6, 33–42, 2025
https://doi.org/10.5194/mr-6-33-2025-supplement
© Author(s) 2025. CC BY 4.0 License.

Open Access
MAGNETIC RESONANCE

Supplement of

Increased sensitivity in electron–nuclear double resonance spectroscopy with chirped radiofrequency pulses

Julian Stropp et al.

Correspondence to: Niels C. Nielsen (ncn@chem.au.dk) and Daniel Klose (daniel.klose@phys.chem.ethz.ch)

The copyright of individual parts of the supplement might differ from the article licence.

S1. Generation of chirp RF pulse waveforms

The waveform of the chirp RF pulse for experiments and simulations is calculated according to

$$\nu_2(t) = \nu_{2,\mathrm{max}} \cdot \cos\left(\int_0^t \nu_{\mathrm{RF}} - \frac{\Delta\nu_{\mathrm{chirp}}}{2} + \frac{\Delta\nu_{\mathrm{chirp}}}{t_{\mathrm{chirp}}}\tau d\tau\right) \cdot a(t) \tag{S1}$$

with

$$a(t) = \sin\left(\frac{\pi t}{2t_{\mathrm{rise}}}\right) \quad \text{for } t < t_{\mathrm{rise}}, \tag{S2}$$

$$a(t) = \sin\left(\frac{\pi(t_{\mathrm{chirp}} - t)}{2t_{\mathrm{rise}}}\right) \quad \text{for } t > t_{\mathrm{chirp}} - t_{\mathrm{rise}}, \tag{S3}$$

$$a(t) = 1 \quad \text{else.} \tag{S4}$$

Table S1. Definition of parameters for chirp pulses and values used in simulations and experiments. *Amplitudes obtained from nutation experiments on ^{14}N at 23.9 MHz.

Parameter	Symbol	Value used in simulations	Value used in experiments
Center frequency	ν_{RF}	4 - 24 MHz	5 - 95 MHz
Bandwidth	$\Delta\nu_{\mathrm{chirp}}$	0.062 - 8 MHz	0.062 - 8 MHz
Amplitude*	$\nu_{2,\mathrm{max}}$	100 & 1000 kHz	140 kHz (500 W) & 62 kHz (100 W)
Pulse length	t_{chirp}	40 µs	10 - 200 µs
Rise/Fall time	t_{rise}	0.2 µs	0.2 µs
Sampling rate	ν_S	2.4 GHz	2.4 GHz
Vertical resolution	r_v	16 bit	16 bit

S2. Setup and Optimization of RF chirp pulses in ENDOR experiments

The first parameter to choose is the bandwidth of the chirp RF pulse and therefore the resolution desired in the experiment (e.g. 1 MHz chirp bandwidth corresponds to approximately 1 MHz potential resolution in the ENDOR spectrum as visible in Figs. 2 and 3a). As a second step the pulse length should be optimized such that the spectral power density is sufficient to achieve an adiabatic passage for spin packets with resonance frequencies within the excitation bandwidth. This is achieved when, the peak intensity does not increase anymore with increasing pulse length and depends on the available RF amplifier output power, the ENDOR resonator and the combined frequency response of the RF chain. In our case, for a 100 W RF amplifier output power and a Bruker X-band MD4 ENDOR resonator an RF pulse length of ca. 100 µs was sufficient for maximum sensitivity and full inversion of all coupled nuclei at 1 MHz chirp bandwidth (see Fig. 2d). We also recommend shaping the pulse edges with quarter sine waves to remove wiggles in the excitation profile. Even though plenty of information for optimal chirp RF pulses

can be found in NMR literature; (Baum et al., 1985; Kupce and Freeman, 1996; Garwood and DelaBarre, 2001) the frequency response of the ENDOR RF circuit, which is often not known precisely and differences between spectrometer setups, renders experimental testing of the optimal pulse parameters more straightforward and faster for many users than an optimization based on calculations.

S3. Supplementary tables

Table S2. g-tensor for CuTPP as published by Brown and Hoffman (1980).

Orientation	x	y	z
g	2.045	2.045	2.190

Table S3. Hyperfine couplings and nuclear quadrupolar couplings for CuTPP for the central Cu ion (in linear frequency units), the four chemically equivalent nitrogen atoms and pyrrole protons as published by Brown and Hoffman (1980).

Nucleus	A_x/MHz	A_y/MHz	A_z/MHz	P_x/MHz	P_y/MHz	P_z/MHz
^{63}Cu	-102.7	-102.7	-615			
^{14}N	54.213	42.778	44.065	-0.619	0.926	-0.307
^{1}H	2.5	0.70	0.80			

S4. Supplementary figures

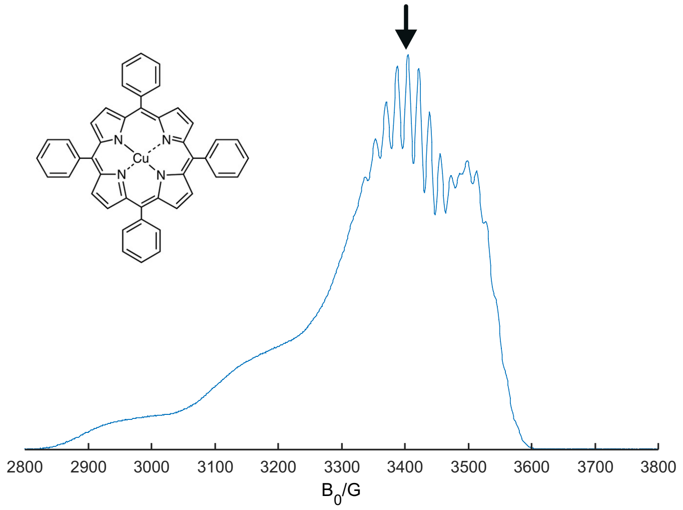

Figure S1. Echo-detected EPR spectrum of CuTPP at 9.78 GHz with an arrow marking the magnetic field position used for ENDOR experiments. For acquisition a Hahn echo sequence with 10/20 ns pulses and a τ of 420 ns was used with a 2-step phase cycle and a shot repetition time of 20 ms. The inset shows the structure of CuTPP.

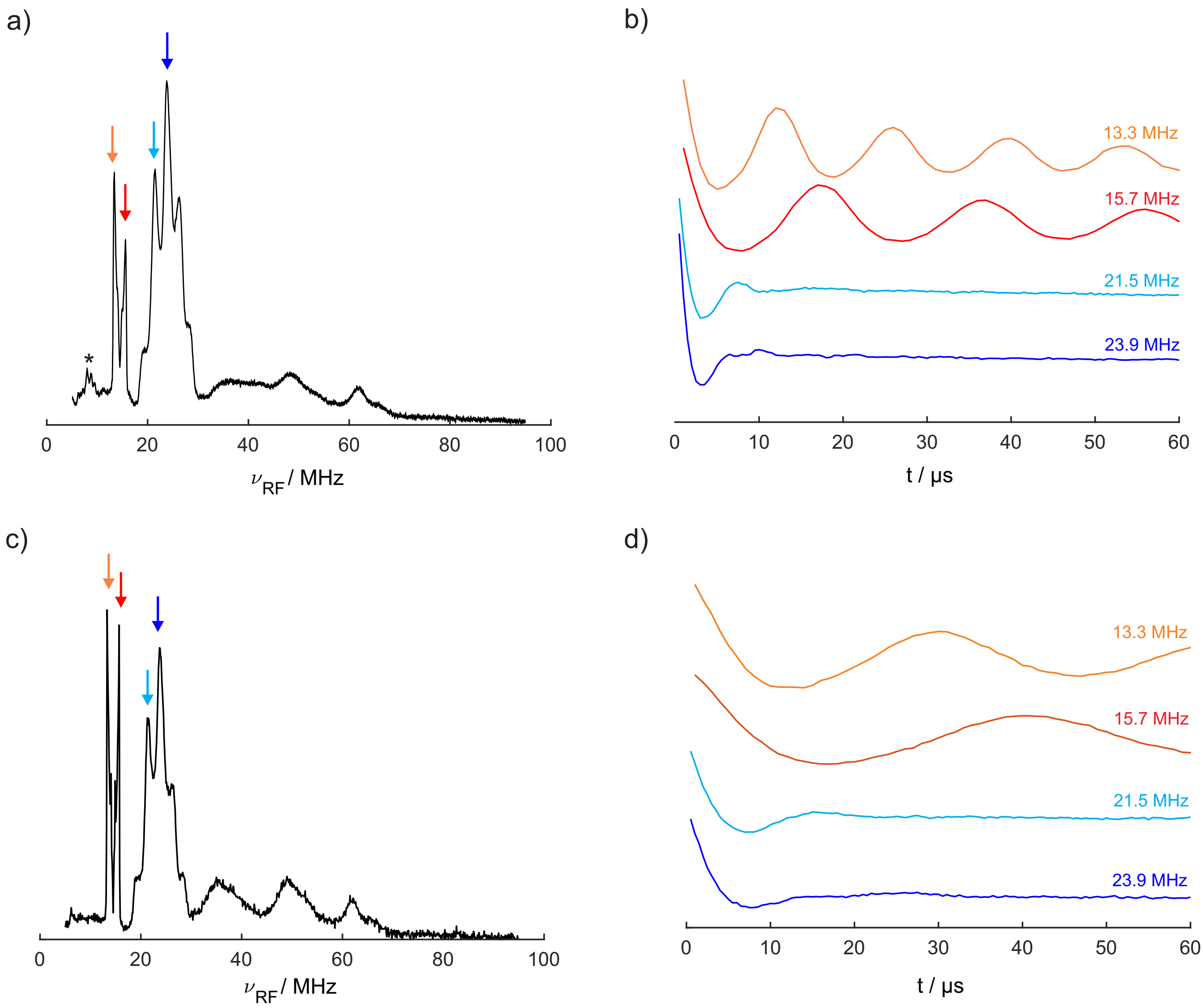

Figure S2. a) Davies ENDOR spectrum of CuTPP with a 500 W single frequency RF pulse of 3.5 µs length; $*^{14}N$ amplifier overtones. b) Rabi oscillations of four selected positions in the ENDOR spectrum. c) Davies ENDOR spectrum of CuTPP with a 100 W single frequency RF pulse of 18 µs length. d) Rabi oscillations of four selected positions in the ENDOR spectrum.

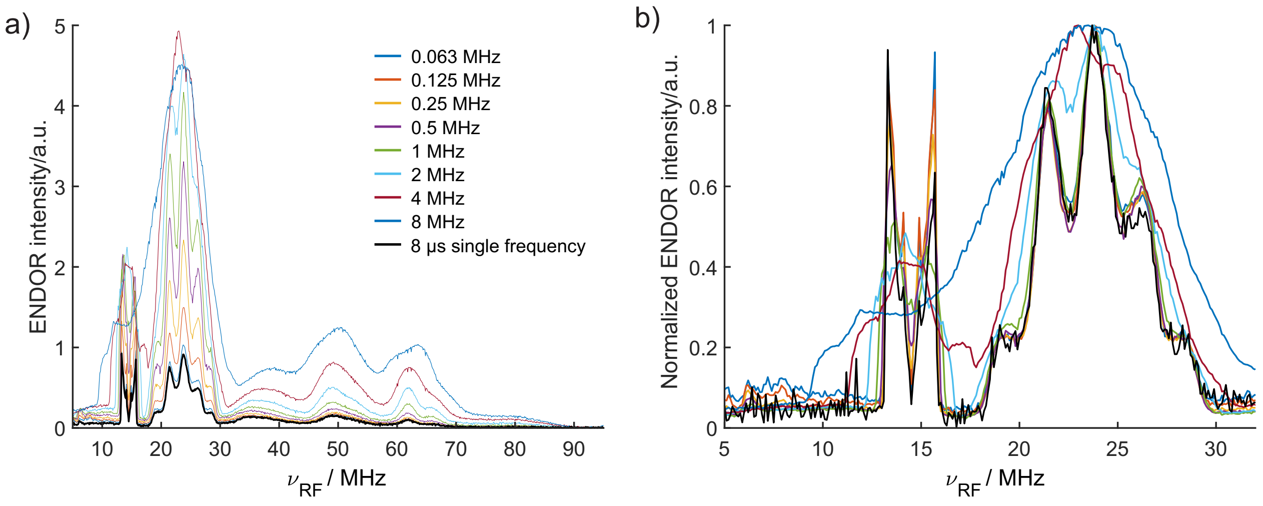

Figure S3. Davies ENDOR spectra of CuTPP with 80 µs chirped RF pulses of different bandwidths compared to a single frequency (sf) ENDOR spectrum recorded with the same RF power (100 W): a) Absolute ENDOR intensity, b) Normalized ENDOR intensity.

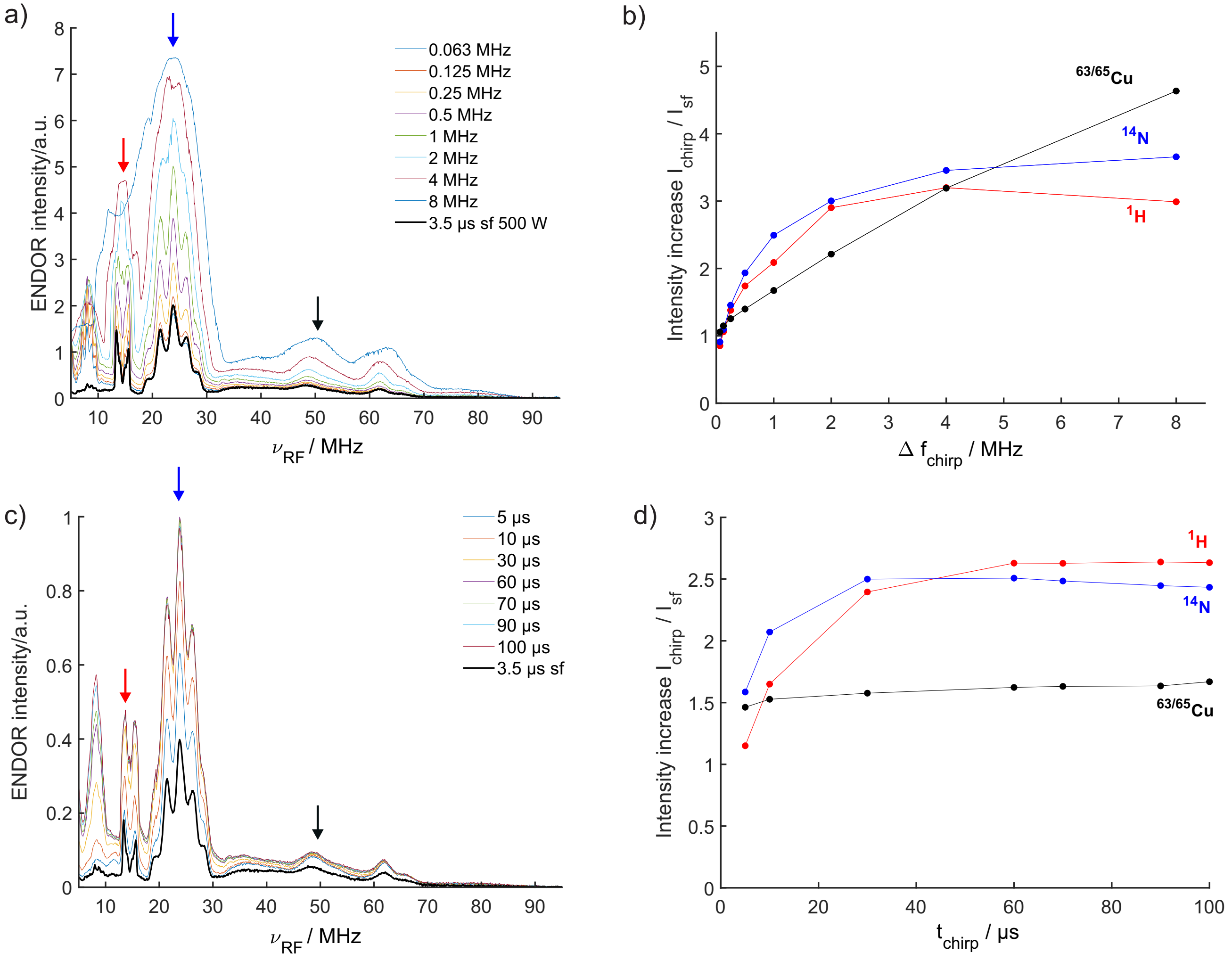

Figure S4. Davies ENDOR spectra of CuTPP with chirped RF pulses of (a) different bandwidths (80 µs pulse length) and (c) different pulse lengths (1 MHz bandwidth) compared to a single frequency (sf) ENDOR spectrum recorded with the same RF power of 500 W. b) and d): Relative ENDOR intensity increase of the largest ^{1}H, ^{14}N and ^{63,65}Cu peaks in chirp ENDOR spectra compared to single frequency ENDOR spectrum (100 W) for different RF bandwidths (b) and different RF pulse lengths (d).

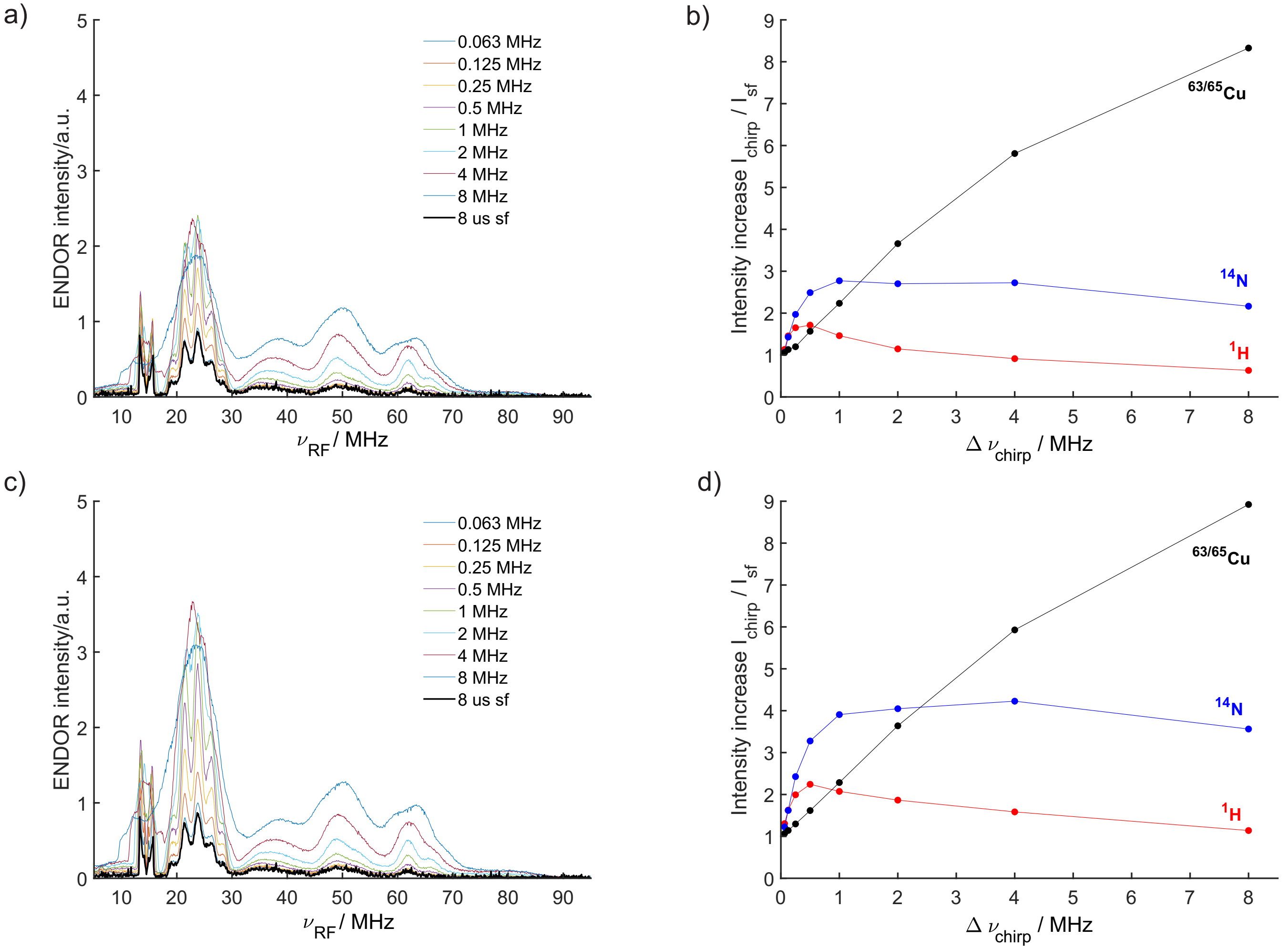

Figure S5. Davies ENDOR spectra of CuTPP with chirped RF pulses of different bandwidths with (a) 20 μs pulse length and (c) 40 μs pulse length using an RF power of 100 W. b) and d): Relative intensity increase of the largest ^{1}H, ^{14}N and $^{63,65}Cu$ peaks in chirp ENDOR spectra for 20 μs (b) and 40 μs (d) compared to single frequency ENDOR spectrum (100 W).

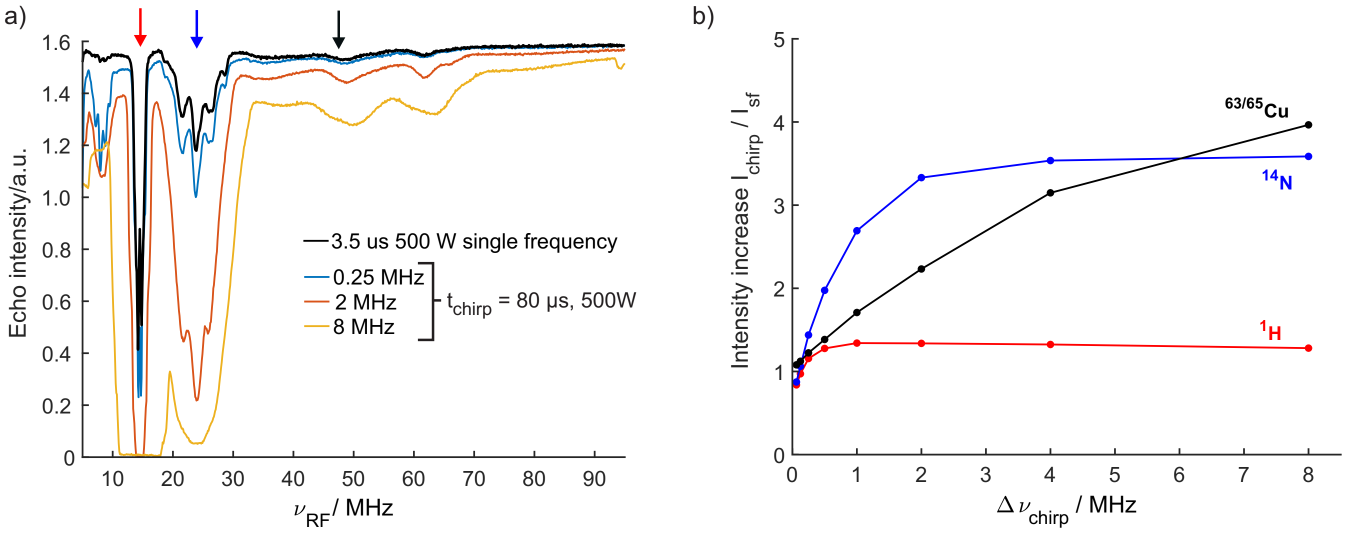

Figure S6. a) Mims ENDOR spectra of CuTPP with 80 μs chirped RF pulses of different bandwidths compared to a single frequency (sf) ENDOR spectrum recorded with the same RF power (500 W). b) Relative intensity increase of the largest ^{1}H, ^{14}N and $^{63,65}Cu$ peaks in chirp ENDOR spectra using different RF-chirp bandwidths compared to to the single frequency ENDOR spectrum (500 W).

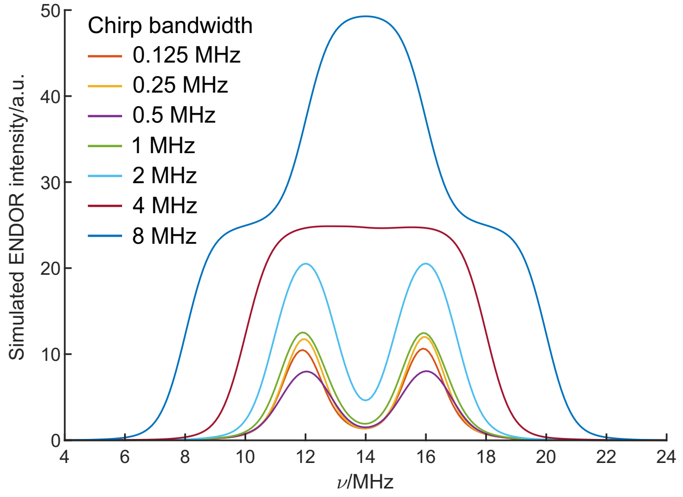

Figure S7. Simulated chirp ENDOR spectra for different chirp bandwidths of an electron-proton 2-spin system with a Gaussian distribution of isotropic hyperfine couplings ($\sigma = 0.5$ MHz). The chirp pulse has a length of 40 μs with 200 ns quarter sine wave weighted edges and an RF amplitude of $\nu_{2,\max} = 1000$ kHz.

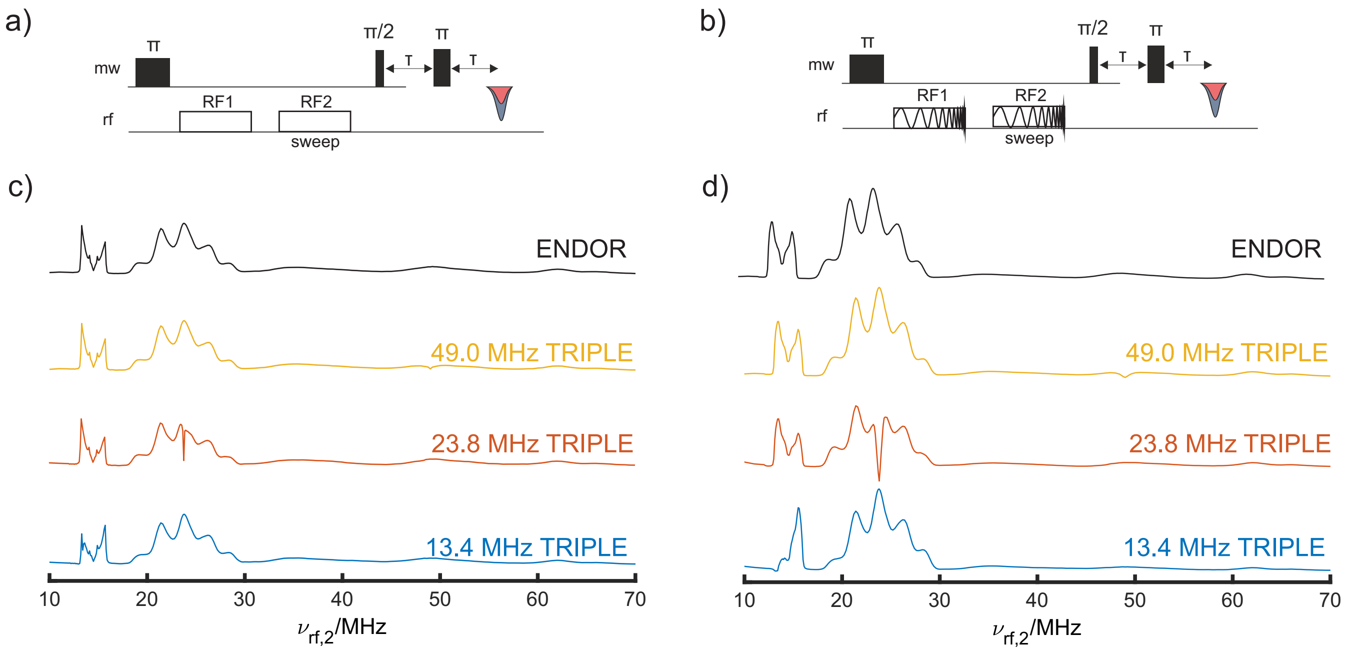

Figure S8. TRIPLE pulse sequences with (a) single frequency RF pulses and (b) chirped RF pulses. Selected TRIPLE traces and ENDOR spectrum of CuTPP with (c) single frequency RF pulses and (d) chirped RF pulses.
